# Supplementary material for: Are carbon emissions trading and green financial instruments synergistic? -Comprehensive quantitative research based on content analysis
Source: PLoS One. 2024 Mar 7;19(3):e0298601. doi: 10.1371/journal.pone.0298601 (PMC10919601; doi:10.1371/journal.pone.0298601)
Supplement: S1 Data — (ZIP) [file pone.0298601.s001.zip › Data/地级市Z_F工作报告.zip!/╔┬╬≈/╬≈░▓╩╨/└┤╫╘╣1⁄2╓┌║┼í╛┬φ┐╦╩2╛▌═°í┐_2014.docx]

各位代表：

现在，我代表市人民政府向大会作工作报告，请予审议，并请各位政协委员和其他列席人员提出意见。

一、2013年工作回顾

过去的一年，面对极为复杂的经济形势，我们在省委、省政府和市委的坚强领导下，全面贯彻落实党的十八大和十八届二中、三中全会精神，牢牢把握稳中求进的工作总基调，以“五项重点工作”为抓手，全力以赴稳增长、调结构、促改革、惠民生，较好地完成了市十五届人大三次会议确定的目标任务。

（一）综合施策重点突破，全市经济呈现出稳中有进、发展向好的可喜局面。面对经济下行压力持续加大的严峻挑战，我们始终把稳增长放在经济工作的首位，及时研判、主动作为，相继出台了加快发展服务业、旅游业和支持小微企业、民间投资健康发展等一系列政策措施，有力地促进了经济平稳较快发展。全年完成生产总值4884.1亿元，增长11.1%。地方财政一般预算收入突破500亿元，增长26.5%，较2010年实现翻番，提前两年完成“十二五”目标。新增中小微企业1.6万户，非公有制经济实现增加值2548.1亿元，占GDP比重达到52.2%。特别是在15项主要经济指标中，我市有8项增速位居15个副省级城市第一，3项位居第二，综合经济实力进一步提升。

（二）狠抓工业强本固基，支撑长远发展的产业基础更加坚实。不断强化“功成不必在我”的理念，致力于打基础、利长远，始终把强工业作为稳增长、调结构的重中之重，坚持创新驱动、政策撬动、项目带动和市场推动，工业突破取得明显成效。全市规模以上工业增加值完成1265.6亿元，增长15.4%。渭北工业区建设加快推进，入区企业达到1204家，实现规模以上工业总产值1013.2亿元，占全市工业比重达到22.5%。三星电子存储芯片工厂如期建成并开始试运行，引进配套企业59户。比亚迪扩建等170个重大工业项目顺利推进，美国强生、韩国SK等全球500强企业相继落户或增资，全年完成工业投资868.6亿元，增长50.2%；新增规模以上工业企业132户、国家级高新技术企业109户，超额完成年度目标任务。

（三）稳定投资扩大消费，拉动增长的方式更趋合理。一年来，我们坚持以项目带投资、以投资促发展，着力在优化投资结构、发挥民间资本作用、提高资金使用效益上下功夫。505个市级重点项目共实现投资1897亿元，完成年计划任务的126%，其中339个产业类项目完成投资1022亿元，是上年的1.6倍。全年完成城建投资297.5亿元，增长18.2%。民间投资完成2566.3亿元，增长21.6%，占全市固定资产投资的50.8%。针对消费市场增长趋缓的问题，我们及时出台了《保持消费市场平稳可持续发展的若干意见》和促进旅游业发展的具体措施，临潼国家旅游度假区、南门区域综合改造提升等重大旅游项目进展顺利，汉长安城未央宫遗址保护展示区免费开放，全年接待海内外游客首次“破亿”，达到1.013亿人次，实现旅游业总收入811.4亿元，分别增长27%和24%。成功举办了欧亚经济论坛、国际通航大会等规模以上展会活动170个，成交额达1173亿元，荣获“2013中国十佳品牌会展城市”称号。华南城、赛格国际购物中心等一批大型商贸综合体投入运营，新建“万村千乡市场工程”农家店130个，覆盖了全市80%以上的行政村。新增限额以上商贸企业217户，是年初目标任务的2.7倍。全市社会消费品零售总额增长14%。

（四）统筹城乡稳农富民，区县经济在协调发展中实现新提升。按照“一类一策”、“分类推进”的原则，加大对区县的支持力度，市财政专项列支2.4亿元用于3个副中心城市和3个县加快基础设施建设。外围区县发展的速度明显加快，主要经济指标增速一路领跑，占全市经济总量的比重持续提升，形成了外围区县与中心城区竞相发展的良好格局。7个城市片区、5个城市组团、9个重点示范镇和4个文化旅游名镇建设步伐加快，启动了47个新型农村社区建设，20.05万人实现“农转居”，“绿色城乡统筹”成为全国示范。全市粮食生产实现“十连丰”，秦岭北麓西安都市现代农业示范区启动建设，新增市级以上现代农业示范园24个、产业化龙头企业37家。扶贫开发工作稳步推进，实施移民搬迁2.09万人，11.6万贫困人口实现脱贫，全面消除绝对贫困。

（五）建管并重完善功能，城市综合承载力不断增强。深入实施缓堵保畅工程，地铁1号线开通运营、3号线进展顺利、4号线全面开工，东门立交建成通车，45条干道支路、55处路段、交叉口和55处公交港湾的改造任务如期完成。新建公共停车位10863个，新增公交车224辆、公共自行车8865辆。城东交通枢纽投入使用，西咸北环线、西临高速改扩建等一批重大交通项目积极推进。10项水源工程建设全面启动，李家河水库大坝浇筑基本完成，新增城市日供水能力5万吨。开工建设1座750千伏和2座330千伏变电站，新建和增容改造110千伏变电站12座，新增变电容量100万千伏安。扎实推进城市精细化管理，城市综合管理数字平台建设和南二环线网落地工程基本完成，占道施工围挡、道路限速不合理等市民反映强烈的城市管理问题得到有效治理，全面完成了20项年度重点整治任务。城棚改工作顺利推进，完成了20个城中村、8个棚户区征收搬迁，回迁安置群众4.29万人。荣获“2013中国形象最佳城市”称号。

（六）生态环境持续改善，美丽西安建设取得新成效。认真落实《西安市秦岭生态环境保护条例》，高标准实施浅山区生态环境建设9项重点任务，秦岭终南山世界地质公园顺利通过联合国教科文组织的中期评估。深入开展国家生态园林城市和国家森林城市创建工作，清凉山等6个公园和60个绿地广场建成开放，长安大道等一批城市绿带建设加快推进，全年新增城市绿地857万平方米，造林7.8万亩。大力实施“八水润西安”工程，完成渭河堤防建设和城市段景观绿化任务，浐灞国家生态湿地公园、沣河生态景区（一期）建成开放，全市新增湿地面积8660亩、生态水面4035亩。加大渭河、皂河污染治理力度，完成新建和提标改造污水处理厂13座，新增污水日处理能力24.5万吨。二氧化硫、化学需氧量、氨氮和氮氧化物排放分别削减3.23%、2.95%、3.05%和5.04%，全面实现年初预期目标。

这里需要特别指出的是，去年以来我市在大气污染治理方面，见事早、行动快、投入大、措施实，率先在全国提出并实施了治污减霾工作，并将其列为环境治理和民生改善的头号工程，市财政累计投入专项资金14.95亿元，拆除燃煤锅炉593台，淘汰黄标车和老旧车辆5.62万辆，关闭了主城区所有造纸企业。全年空气质量按新标准统计，优良天数达到138天，超出省考指标33天。

（七）深化改革力促开放，发展的内生动力持续增强。切实推进简政放权，下放行政事权71项。综合治税工作全面启动，“营改增”试点进展顺利。农村产权制度改革试点全面推开，流转土地46.6万亩。医疗联合体及县镇村医疗服务一体化改革初显成效。“大学区管理制”实现全覆盖，改革经验在全省推广。扎实推进统筹科技资源改革，投入20.7亿元布局建设了云计算应用等一批新兴产业创新基地，全年实现技术成果交易额415亿元、增长38.5%，本地企业吸纳技术成果交易额突破100亿元，荣获“全国十大创新型城市”称号。

积极推进丝绸之路经济带建设，联合沿线国内外15个城市共同签署了《西安宣言》，大唐西市丝绸之路风情街（一期）开街运营，与中亚城市马雷、撒马尔罕建立友好关系，“长安号”国际货运班列正式开通，西安的国际影响力进一步提升。全年实际引进内资1430.3亿元、实际利用外资31.3亿美元，分别增长19.1%和26.3%。实现服务外包合同额8.97亿美元，增长64.5%。

（八）关注民生强化保障，人民生活进一步改善。全年财政一般预算支出的84.4%和新增财力的88.8%用于民生，总计达到615.6亿元，增长25.8%，人民群众享受到了改革发展的更多实惠。

“10项惠民实事”全面完成。新开工保障性住房8.78万套，基本建成6.8万套。完成农村危房改造4205户，181万职工纳入住房公积金制度保障体系，占全市职工的70%。新增集中供热面积3243万平方米，新增天然气居民用户20万户。创建放心食品店、蔬菜直销点570个。解决了农村29万人饮水安全问题。学前教育三年行动计划全面完成，累计新建、改扩建标准化幼儿园286所。校车工程试点范围进一步扩大，享受营养改善计划和蛋奶工程学生人数达到32.97万人。建成了40个农村养老互助院和100个老年餐桌示范点，完成了100个村和100个社区养老服务试点任务。城乡居民大病保险工作正式启动，城镇基本医疗保险参保率、新农合参合率分别达到98.5%和99.1%。新殡仪馆如期建成并投入使用。

各项社会事业不断进步。全年城镇新增就业12.58万人，城镇登记失业率为3.4%，农村劳动力转移就业100.5万人，城乡居民收入分别达到33100元和12930元，增长10.4%和13%。为解决城乡低收入家庭生活困难，制定出台了13项具体帮扶措施，新增财政支出3.4亿元，惠及群众30万人。文化惠民工程深入推进，“博物馆之城”建设三年目标提前完成，累计建成各类博物馆100座，组织文化活动、惠民演出1400多场。新建、更新社区全民健身路径100个，建成农民健身工程700个，成功举办第15届市运会。精神文明建设成效显著，4人荣获全国道德模范称号，11人登上中国好人榜。“平安西安”建设不断深化，“五防五打五管控”工作扎实有效，多元化矛盾纠纷化解体系进一步完善，安全生产形势保持稳定。再次荣获“中国最具幸福感城市”称号。

（九）正风肃纪转变作风，政府自身建设不断加强。深入开展党的群众路线教育实践活动，坚持问题导向，立说立行，积极回应社会关切，查处解决了一批教育乱收费、环境违法和违规销售经适房等问题。在全市政府系统集中开展作风纪律整顿活动和“九个专项治理”行动，出台了严禁公款吃喝等“十条禁令”，庸懒散奢等不良风气得到有效整治，全市“三公”经费支出下降8.5%，市政府文件、会议分别减少25.9%和37.5%。深入推进政务公开，积极开展大学生暑期到政府机关见习和公民代表进政府活动，人民网网民留言回复率达99.4%，被授予“全国网民留言办理工作示范城市”。全年向市人大常委会提交地方性法规草案7件，办理人大代表建议296件，政协提案786件。出台《西安市行政程序规定》，基本建成四级政务服务体系。认真落实“一岗双责”，加大审计力度，政府廉政建设和反腐败工作扎实推进。

过去一年，我市其他方面的工作也取得了明显进步。军民融合、双拥共建活动深入开展，计划生育、妇女儿童、老龄、残疾人等事业加快发展，普法宣传、民族宗教、参事、统计、史志档案、社科、人防、气象、防震减灾、慈善、红十字会等工作都取得了新成绩。

各位代表！回顾过去一年的工作，我们所取得的一切成绩，是省委、省政府和市委正确领导的结果，是市人大、市政协和社会各界有效监督、大力支持的结果，是全市人民团结拼搏、共同奋斗的结果。在此，我代表市政府，向全市人民，向中、省驻市单位，向驻西安部队和武警官兵，向所有关心支持西安建设发展的海内外朋友，表示衷心的感谢！

在总结成绩的同时，我们也清醒地看到，工作中还存在不少困难和问题。主要表现在：经济发展中一些结构性、深层次的问题尚未有效解决，工业稳升向好的基础仍不牢固，现代服务业发展相对滞后，非公经济规模和质量有待进一步提升；经济外向度和国际化水平与建设丝绸之路经济带新起点的定位还有很大差距；城乡发展不均衡、公共服务不均等的问题仍然突出，群众对环境污染、交通拥堵、食品安全等民生问题意见还比较大；政府职能转变还不到位，简政放权力度需要进一步加大，勤政廉政建设和反腐败斗争仍是长期任务。对此，我们一定要采取切实措施，认真加以解决。

二、2014年工作安排

今年是全面深化改革的开局之年，也是实现“十二五”规划目标的关键一年，更是丝绸之路经济带新起点建设的起步之年。面对世界经济仍然错综复杂、我国经济下行压力依然较大的严峻局面，我们务必保持清醒头脑，准确把握形势，坚定信心，抢抓机遇，积极作为，确保我市经济社会平稳健康发展，努力当好“三个陕西”建设的排头兵。

根据市委十二届四次全会的安排部署，今年政府工作的总体要求是：以党的十八大和十八届三中全会精神为指导，以全面深化改革为统领，认真贯彻落实省委、省政府和市委的各项决策部署，坚持稳中求进、改革创新、提质增效的工作基调，围绕“建设丝绸之路经济带新起点”和“社会和谐、城市宜居”的新要求，以“五项重点工作”为抓手，全力稳定增长，着力调整结构，切实优化环境，积极扩大开放，持续改善民生，努力打造西安经济升级版，不断开创全面建设具有历史文化特色的国际化大都市的新局面。

综合各方面因素，建议今年全市经济社会发展的主要预期目标是：生产总值确保增长11%以上，地方财政一般预算收入增长13.5%以上，城镇登记失业率控制在4.5%以内，城乡居民收入分别增长11%和12%，物价涨幅控制在4%左右，社会消费品零售总额增长13.5%以上，全社会固定资产投资增长20%以上，万元GDP能耗下降3.3%，二氧化硫、氮氧化物、化学需氧量和氨氮排放分别削减0.5%、3%、1.7%和2.2%。

为实现上述目标，要重点做好以下八方面工作。

（一）全面深化改革，进一步增强发展内生动力。

认真贯彻落实中央全面深化改革的决定和省委的实施意见，以及市委《关于全面深化城市管理和社会治理改革的若干意见》，着眼于充分发挥市场配置资源的决定性作用，坚持把改革创新贯穿于经济社会发展的各个领域各个环节，力争在重点领域和关键环节上取得新突破。

深入推进重点领域改革。推动国有资产监管从管资产为主向管资本为主转变，加快培育有实力的国有资本公司。积极引入非公有资本参与市属国有企业产权多元化改革，大力发展混合所有制经济，加快推进西纺集团与五环集团重组整合。扎实做好“营改增”扩围试点。全面开展农村宅基地、集体建设用地使用权确权登记发证工作，加快建立农村产权流转交易市场。鼓励、支持各类金融服务组织向农村延伸，年内实现小额贷款公司在涉农区县全覆盖。进一步加强对各级政府融资平台的监管，严格政府举债程序，有效规避政府性债务风险。不断创新城市管理体制机制，坚持重心下移、属地管理，确立区级政府在城市管理中的主体地位，切实解决“看到管不到、管到看不到”的问题。

加快推进统筹科技资源改革。支持高新区建设国家自主创新示范区、沣东新城建设国家统筹科技资源改革示范基地，加快阎良国家级创新型产业集群和碑林环大学创新产业带建设。围绕打造支柱产业，构建产业、科技、金融深度融合，企业、高校、院所有机结合，研发、中试、生产、服务环环相扣的科技创新链条。提升科技大市场服务效能，推进“高校院所人才服务企业工程”，促成更多科技成果加速转化、就地转化，力争年内全市技术成果交易额突破500亿元。

加强社会治理创新。以和谐社区建设为重点，加大老旧社区改造力度，健全完善社区基础设施和服务功能，全面推行网格化管理、社会化服务。强化企事业单位、人民团体在社会管理和服务中的职责，引导社会组织健康有序发展，支持和发展志愿服务组织。健全“三调联动”体系，建立调处化解矛盾纠纷综合机制，推进信访制度改革，完善网上信访受理，及时协调和解决人民群众的合理诉求。坚持源头治理、系统治理、综合治理、依法治理，着力在更高起点上推进平安西安建设。继续深化“大学区管理制”改革，促进公办学校校长、教师交流轮岗，加快现代教育信息技术建设与应用，努力缩小区域、城乡、校际差距。全面放开县城和建制镇的户籍限制，消除人口城镇化的制度阻碍。

（二）持之以恒强工业，不断夯实稳升向好的发展基础。

坚持把强工业作为调整产业结构的主攻方向，以各级各类工业聚集区和重大工业项目为抓手，深入实施工业突破战略，力争全年规模以上工业增加值增长15%以上。

加快推进渭北工业区建设。坚持一手抓基础配套，一手抓招商引资。加快建设渭北大横线、泾河引水等重大基础设施，着力抓好兵器产业基地、中航工业园等重点产业园区发展，力争工业投资超过400亿元，增长50%以上。围绕各组团主导产业定位，加速形成产业聚集，积极争取现代汽车项目落户我市。确保规模以上工业增加值增长17%以上。

继续实施“工业投资倍增计划”和“工业企业成长工程”。着力抓好196个市级重点工业项目，确保三星电子、比亚迪二期等70个项目年内建成投产，全市工业投资增长45%左右。强化对55户产值超10亿元重点企业的政策支持和服务保障，加大对中小微企业的扶持力度，全年新增规模以上工业企业130户。切实发挥市级专项资金的撬动作用，重点对企业技术改造、技术创新、节能降耗等予以扶持，积极引导土门、幸福路等主城区的工业企业退城入区、转型发展。深入实施质量强市战略，积极创建全国质量强市示范城市。

大力发展战略性新兴产业。持续放大“三星效应”，带动电子级硅材料、集成电路设计、半导体封装等产业发展，重点打造千亿元智能手机产业集群，加速形成完整的电子信息产业体系。加快国家下一代互联网示范城市和“智慧城市”建设，促进信息化、工业化深度融合。以我市获批全国新能源汽车示范推广城市为契机，依托比亚迪、陕重汽新能源汽车项目，推动传统汽车产业转型升级。积极跟进大飞机研制生产，协力推进“新舟700”飞机研发，着力抓好卫星应用产业聚集区建设，加快发展航空航天产业。深入实施“科技企业小巨人”培育计划，重点培育300家创新能力强、成长速度快、市场占比大的科技型骨干企业，力争全年高新技术产业实现增加值700亿元。

（三）推动服务业转型升级，积极扩大社会消费。

立足我市资源禀赋和比较优势，加快改造提升传统服务业，大力发展现代服务业，充分激发消费市场活力，不断提升第三产业对全市经济的贡献率。

全力打造现代服务业聚集区。重点抓好8个国家级和10个省、市级服务业综合改革试点聚集区建设。加快推进区域性金融中心建设，争取上合组织开发银行落户西安，力促更多中外银行在我市设立分支机构，鼓励民营资本兴办中小银行。以科技服务业为支撑，带动生产性服务业向高端发展，重点培育50家研发、测试等科技服务机构。依托西北金属、广汇汽车等重点物流园区，推动制造业与物流业联动发展。年内新增规模以上服务业企业100家，服务业增加值增长9.5%以上。

推动旅游文化产业加快发展。切实用好促进旅游业发展的各项政策，着力抓好秦岭终南山世界地质公园、阎良航空旅游度假区等一批重大旅游项目建设，国庆节前南门景区和环城文化休闲体验区建成开放。积极发展休闲、商务等新兴旅游业态，完善自助游、自驾游服务体系，不断优化旅游产品结构。加快西安火车站游客集散中心等综合平台建设，在市、区两级建立旅游综合协调机制、联合执法监管机制和投诉统一受理机制，深入整治旅游市场秩序，力争年接待海内外游客1.2亿人次，实现旅游业总收入950亿元以上。积极推动文化产业与旅游、科技、金融等产业融合发展，引导更多市场要素向文化产业聚集，加快建设国家数字出版基地、印刷包装产业基地和广告产业园，支持各类文化企业互动发展，促进龙头企业跨行业、跨所有制兼并重组，着力提升我市文化产业的整体实力和竞争力。年内新增文化企业400家，全市文化产业增加值达到525亿元，增长25%。

积极营造良好消费环境。扎实推进七大城市片区开发，加快东大街、解放路等传统商圈改造提升，启动建设长安路中央商务区等新兴商圈，积极发展地铁经济。大力培育休闲、健康、信息等新的消费热点，扩大服务性消费在居民消费中的比重。深入推进“万村千乡市场工程”和“镇超工程”，进一步优化农村消费环境。以创建国家电子商务示范城市为契机，支持中西部商品交易中心、京东商城等龙头企业运营发展，吸引更多电商巨头落户西安。

（四）以优化结构为着力点，不断提高投资的质量和效益。

坚持以项目带投资、以投资促发展的思路不动摇，在保持投资合理增长的同时，更加注重优化投资结构，充分释放民间资本的活力，努力在提高投资质量和效益上实现新突破。

全力抓好重点项目建设。按照“保续建、保重点、保民生、利长远”的要求，引导各类资金更多投向结构调整、基础设施、民生改善、环境保护等领域。市级重点建设项目年度完成投资1850亿元，力争产业类项目投资占比达到60%以上，确保60个重点项目建成达效。全年完成城建投资352亿元。

着力构建多元化投融资格局。认真落实国务院“新36条”和我市《关于鼓励和引导民间投资健康发展的意见》，鼓励民间资本进入金融、交通、水利、市政、教育、医疗等六大领域，促进民间投资持续健康增长。加强银、政、企合作及项目对接，支持引导信贷投放向重点领域、重点项目倾斜。深入实施“双推双增”融资工程，推进多层次资本市场建设，支持企业在“新三板”挂牌交易筹集资金。全年实现社会融资2300亿元以上。

扎实做好招商引资工作。围绕主导产业和配套产业链，大力引进世界500强、国内500强企业和战略投资者，推动国内外大型企业在我市建立区域总部、研发中心和生产基地。不断创新招商方式和手段，积极开展针对重点国家、地区的招商推介活动。完善招商项目保障、推进和考核机制，确保签约项目顺利落地，力争全年实际引进内外资分别增长15%和25%以上。

（五）积极实施开放带动战略，着力打造内陆开发开放新高地。

抓住国家推进丝绸之路经济带建设的战略机遇，加快对外开放环境建设，积极承接产业转移，不断增强区域中心城市的开放功能，全面提升我市的经济外向度和国际化水平。

全面启动丝绸之路经济带新起点建设。抓紧落实我市《实施方案》，围绕“一高地六中心”建设，完成相关规划编制工作。积极构建以西安为中心的综合立体交通体系和国际中转枢纽港，年内开通西安至哈萨克斯坦国际航线，尽快落实72小时过境免签，推进“长安号”国际货运班列常态运行。扎实做好丝绸之路联合申遗和“丝绸之路经济带西安自由贸易区”申报工作。启动丝绸之路文化园、博物馆及起点广场建设，创办“丝路风情”文化系列活动，促进与沿线城市在文化、产业、教育、科研、旅游等多方面的交流。

积极搭建对外开放平台。启动建设欧亚经济论坛综合园区，推进上合组织成员国在我市设立领事馆。支持有实力的企业，参与《上合组织成员国多边贸易合作纲领》确定的10大领域、85个重点合作项目建设。策划实施“西安走进中亚西亚”系列推介活动，参与办好“首届丝绸之路经济带国际博览会”。加快建设一批国际学校、医院和社区，不断完善城市涉外综合服务功能。

大力发展外向型经济。抢抓国家扩大内陆沿边开放战略机遇，加快推进综合保税区建设，重点发展以展示交易、保税物流、出口加工、服务外包为特色的外向型经济。鼓励西安优势企业走出去建立生产基地，策划建设“中亚•长安产业园”。加快电子口岸建设，发展跨境电子商务，积极创建全国跨境电子商务试点城市。

（六）以新型城镇化建设为统领，加快推进城乡一体化发展。

按照生产空间集约高效、生活空间宜居适度、生态空间山清水秀的要求，加快推进以人为核心的城镇化建设，着力提升我市城乡一体化发展水平。

进一步优化城市空间布局。结合我市城市总体规划修改工作，尽快出台《城市规划导则》等一批约束性规划。在明确城市开发边界的前提下，加快推进城市建成区的改造提升，积极实施“南优、北拓、东延、西联”战略，使主城区的城市空间布局更加合理、更加优化。

“南优”，就是依托秦岭北麓生态屏障和曲江新区、航天基地、常宁新城等重要板块，突出功能优化、内涵式发展，着力打造生态优美、宜居宜业的美丽新城南。年内完成植物园迁建工程。加快潏河湿地公园建设。打通城市中轴线南段，实现长安大道与南长安街无阻碍贯通。

“北拓”，就是抓住渭河城市段综合治理、浐灞国家湿地公园建成开放等重要机遇，全面提升绕城高速以北至渭河区域的城市化水平。年内建成灞河入渭口特大桥。启动实施西铜一级公路城市段（渭河以南段）改扩建工程，加快北客站周边区域开发，支持草滩生态产业园发展，协调推进西安至阎良城际铁路建设。

“东延”，就是以国际港务区、纺织城综合发展区、临潼国家旅游度假区为龙头，加快城市公共基础设施向东辐射延伸，促进产城融合，推动城东区域大发展、大跨越。启动建设临潼市域轨道交通，力争秦汉大道年内建成通车。支持曲江、临潼联合打造全国“绿色城乡统筹”重大示范工程。

“西联”，就是借助西咸新区成为国家创新城市发展方式试验区的重大机遇，充分发挥省市共建大西安的政策效应，加快推进西咸一体化步伐，推动城西区域设施共建共享、产业互联互融。支持沣东新城加快昆明池、国际车城等四大板块建设，加快三桥商圈、六村堡片区改造步伐。争取启动1号线咸阳延伸段建设，推进西咸公交无缝对接。

切实提高新型城镇化质量。以有能力在城镇稳定就业和生活的常住人口为重点，深入推进农村居民进城落户，年内转移户籍人口14万人。进一步健全社保、医疗、保障性住房等社会福利跟进机制，突出抓好农村养老保险和医疗保险规范接入城镇社保体系、农民工职业培训和随迁子女免费接受义务教育等工作，努力让所有进城落户的农村居民都能平等享受到完整的市民待遇。以构建科学合理的城镇体系为支撑，加快推进3个副中心城市、5个城市组团、9个重点示范镇和4个文化旅游名镇建设，积极实施县城、镇街扩容改造工程，不断完善城镇综合服务功能，促进产业集聚、人口集中。坚持因地制宜、突出特色，注重文化传承，保留历史记忆，严格建设质量，真正让群众放心、满意，经得起社会和历史的检验。

持续推动县域经济突破发展。不断完善“一类一策”制度，建立差异化扶持政策体系。深化开发区与区县合作共建模式，推动区县错位发展、协同发展。切实抓好“三农”工作，全面落实各项强农惠农富农政策，确保农民收入稳定增长。大力发展都市型现代农业，严格耕地保护，重点抓好粮食高产创建工程，全年培育认定20个省、市级农业示范园，新增农业产业化龙头企业20家、市级示范合作社30个。建立协调统一的市县（区）农产品监管、检测、执法工作机制，将农产品质量安全纳入涉农区县绩效考核范围。扎实推进农村饮水安全工程建设，年内解决20万农村人口饮水安全问题。以建设“美丽乡村”为目标，深入开展农村环境综合整治，完成325个新农村重点村建设任务，创建10个以上生态乡镇和20个以上生态村。认真落实扶贫开发“三年行动计划”，实行精准扶贫，年内完成3.1万移民搬迁和13万贫困人口脱贫任务。

（七）以建设宜居城市为目标，不断优化人居环境。

建设宜居城市，是民之所盼、政之所为。必须把“让群众生活更舒适”作为城市建设管理的价值追求，体现在每一个细节中，不断完善城市功能，提升城市品质。

坚决打好治污减霾攻坚战。认真落实大气污染防治“国十条”和我市《治污减霾工作实施方案》，坚持铁腕治霾，狠抓源头治理，加强联防联控。积极推进“煤改气”、“煤改电”工程，年内确保完成350台燃煤锅炉拆改任务，力争两年内实现主城区无煤化。加大机动车尾气污染治理力度，年内淘汰1.48万辆黄标车和老旧车辆，新增1万辆清洁能源机动车。强化建筑工地扬尘污染治理措施，完善建筑垃圾清运全程监控系统，提高道路保洁水平，努力使城区的扬尘污染得到有效控制。加快西郊热电厂烟气综合治理进度，确保按期达标排放。继续抓好以“两河一区”为重点的水污染治理，加快污水处理厂及配套管网建设，确保渭河干流西安段出境水质稳定达标。

深入实施缓堵保畅工程。进一步完善城市路网结构，实施朱宏路—北二环立交等54个城市交通改善项目，完成环城南路综合改造、太白路—丈八路立交等重点工程，改造、拓宽、打通一批城市道路交叉口、拥堵点和断头路，新建公共停车位8000个。加快地铁建设，确保2号线南段6月底前建成通车，3号线年底实现“洞通”。协调推进西咸北环线等3条省市共建高速公路项目，加快国道310、108和省道107等干线公路改扩建工程。继续加快公交都市建设，开工建设西安北站、城西和城南（二期）综合交通枢纽，合理调整公交线网和车辆配置，在市区主干道和繁华路段新增一批出租车停靠点或的士港湾，着力解决出租车停靠难和市民打车难问题，年内投放公共自行车2万辆，初步形成轨道交通、常规公交、慢行交通无缝衔接的综合服务体系，鼓励市民低碳出行。

坚持不懈抓好城市精细化管理。制定《西安市城市治理条例》，依法对城市规划建设、市政设施、市容环卫、道路交通、生态环境等公共事务和秩序进行综合管理，使城市管理更加制度化、规范化、程序化和精细化。继续加大对交通违法、渣土车、野广告、占道经营等城市管理顽疾的整治力度，加快一体化城市综合管理数字平台建设，不断提升城市管理效能。启动火车站改扩建工程，做好东关地区综合改造前期工作。突出抓好百条标准化管理示范街建设，加快推进“架空线缆”落地，抓紧实施夜景亮化提升工作，积极开展主干线、城市出入口延伸线、广场和旅游景点周边区域的美化工程，努力在“做美城市”上取得新突破。

加快推进美丽西安建设。严格落实《秦岭西安段浅山区保护利用规划实施方案》，重点抓好沿山路景观提升、绿道建设、废弃工矿厂区生态恢复等7项重点工程，依法关停取缔保护区内所有违规采矿企业。加快推进“八水润西安”工程，继续抓好渭河、灞河、黑河等河道水系治理和水源工程建设，年内建成李家河水库，新增湿地面积6080亩、生态水面2500亩，饮用水源水质达标率保持在96%以上。严控水资源管理“三条红线”，大力推进节约用水，切实提高再生水利用率。以减量化和资源化为重点，进一步加强城市垃圾管理，年内开工建设3个生活垃圾处理厂，新增9个建筑垃圾消纳场。继续完善汉长安城大遗址保护区配套服务设施，做好申遗区内的群众安置。深入推进国家生态园林城市和森林城市创建工作，大力实施屋顶绿化、立体绿化和城改边角地绿化，加快建成一批城市林带，全年新增城市绿地面积450万平方米，完成造林6万亩。

全力维护城市安全。牢固树立安全发展意识，进一步落实企业主体责任、职能部门监管责任和基层属地管理责任，做到守土有责、守土尽责，坚决遏制重特大安全事故发生。完善三级食品药品监管体制，加强追溯体系和技术检测能力建设，严厉打击制售假冒伪劣食品药品等不法行为。加强安全生产“三基”建设，强化对地铁、高层建筑、特种设备、市政公用管线等领域的安全管理，加大隐患排查力度，确保公共安全。健全立体化社会治安防控体系，有效预防和依法惩治违法犯罪，切实维护社会稳定。加强防灾预警体系建设，提高气象、地质、地震灾害防御能力，为城市安全增设“防火墙”。

（八）扎实办好惠民实事，持续增进民生福祉。

积极回应人民群众对美好生活的新期待，继续以“10项惠民实事”为重点，扎实推进各项民生工程，切实把财力用到群众最需要的地方，实现发展成果更多更公平地惠及全市人民。

大力实施就业优先战略。鼓励创业带动就业，年内发放创业贷款13亿元，带动就业8万人。进一步强化就业帮扶，重点解决好高校毕业生、困难群众和进城农民的就业问题。以政府购买形式增加的基层公共管理和社会服务岗位，更多用于吸纳高校毕业生就业。力争全年城镇新增就业11万人，农村劳动力转移就业67万人。

着力提升社会保障水平。坚持保基本、兜底线、促公平、可持续的原则，认真落实我市改善低收入家庭生活的13项政策措施，确保每一户困难群众的基本生活都有保障、有提高。按照城乡统一标准提高居民社会养老保险待遇，稳步扩大医疗保险报销范围。加快住房保障和供应体系建设，推进廉租房、公租房并轨运行，年内新开工保障性住房10万套，提供房源4万套。进一步完善城棚改项目周边的基础配套和公共服务设施，完成17个城中村、8个棚户区征收搬迁和2.8万人回迁安置任务。积极应对人口老龄化，加快发展以老年餐桌、老年健康和精神慰藉为重点的老年服务产业，扩大城市社区养老服务和农村幸福院建设的覆盖面，力争全年新增机构养老床位3000张。

推动各项社会事业协调发展。继续加大教育投入力度，启动实施第2期学前教育三年行动计划，加快建设36所开发区学校、幼儿园，完成130所义务教育学校标准化建设任务。持续加大教育乱收费治理力度，切实维护群众利益。健全完善基层医疗卫生服务体系，鼓励县级以上医院托管社区、镇街医疗卫生机构。加快市人民医院、第三医院建设进度，确保中医医院完成搬迁并投入使用。进一步规范医疗服务行为，切实解决群众看病难、看病贵问题。落实“单独二孩”政策，推进“母亲健康工程”和“优生促进工程”。建立区县公共法律服务中心，依法维护公民合法权益。完善基层公共文化体育设施，启动市体育场改造工程，新建、更新社区全民健身路径120个、农民体育健身工程600个。加快推进军民融合，深入开展双拥共建活动。进一步支持工会、共青团、妇联等人民团体发挥桥梁纽带作用，扎实做好民族宗教、广电、妇女儿童、残疾人、参事、人防、社科、史志档案等工作。

三、建设人民满意政府

做好今年工作，关键是要加强政府自身建设。我们要以深入开展党的群众路线教育实践活动为契机，切实改进工作作风，全面推进依法行政，着力增强政府的公信力和执行力。

一是加快转变政府职能。继续做好简政放权工作，坚持“积极稳妥、责权统一”的原则，按照“放得下、接得住、管得好”的要求，分批取消和下放部分市级行政审批事项和城市管理事权。充分发挥市场在配置资源中的决定性作用，激发市场主体的创造活力，切实减少投资、生产经营活动、资质资格许可等方面的行政审批事项，真正把政府工作的重点，转移到创造良好发展环境、提供优质公共服务、维护社会公平正义上来。

二是不断规范公权运行。坚持用制度管权管事管人，推行政府部门权力清单制度，依法公开权力运行流程，让权力在阳光下运行。规范行政执法程序，建立法律顾问制度，全面落实行政执法责任制，做到严格规范公正文明执法。自觉接受人大、政协和社会各界监督，进一步拓宽反映社情民意和群众参政议政的渠道，不断提高政府决策的科学化、民主化、法治化水平。

三是着力提高行政效能。严格落实我市治理“文山会海”的25条控制性措施，全面清理各类评比达标表彰评估项目。按照“严、准、细、实、快”的工作标准，不断加大治庸、治懒、治散力度，强化行政问责和效能监察，启动实施末位查究制，坚决整治行政不作为、乱作为、慢作为等问题，确保各项工作任务落实到位。

四是切实加强反腐倡廉。严格执行中央《党政机关厉行节约反对浪费条例》和国务院“约法三章”，坚持勤俭办一切事业，今年市本级公用经费预算统一压缩5%。全面推进惩治和预防腐败体系建设，加强审计监督，严肃查处涉及领导机关、领导干部滥用职权、贪腐堕落、失职渎职等案件，努力营造风清气正、廉洁高效的政务环境。

每一位政府工作人员都要时刻牢记为民服务的宗旨，时刻牵挂群众的安危冷暖，时刻站在群众立场想问题、做决策，不遗余力地为群众办实事、解难事、谋福祉，始终做到一心为民、克己奉公，始终做到勤政务实、敢于担当，始终做到敬业守责、清正廉洁，以实际行动赢得支持、取信于民。

各位代表，全面深化改革的号角已经吹响，新的目标催人奋进。让我们紧密团结在以习近平同志为总书记的党中央周围，在省委、省政府和市委的坚强领导下，抢抓机遇，真抓实干，奋力拼搏，为建设社会和谐、城市宜居、人民幸福的新西安而努力奋斗！
